# Editorial: New insights into pediatric neurology: neurological disorders and epileptic encephalopathies

**DOI:** 10.3389/fneur.2026.1812862

**Published:** 2026-03-11

**Authors:** Carla Consoli, Giulia Spoto, Antonio Gennaro Nicotera, Gabriella Di Rosa, Sebastiano Antonino Musumeci

**Affiliations:** 1 Unit of Child Neurology and Psychiatry, Department of Human Pathology of the Adult and Developmental Age “Gaetano Barresi”, University of Messina, Messina, Italy; 2 Unit of Child Neurology and Psychiatry, Department of Biomedical Sciences, Dental Sciences and Morphofunctional Imaging, University of Messina, Messina, Italy; 3 IRCCS Centro Neurolesi Bonino-Pulejo, Messina, Italy; 4 Unit of Child Neurology and Psychiatry, Maternal-Infantile Department, University of Messina, Messina, Italy; 5 Oasi Research Institute-IRCCS, Troina, Italy

**Keywords:** autoimmune encephalopathy, cannabidiol, developmental and epileptic encephalopathy, epilepsy, genotype–phenotype heterogeneity, ketogenic diet, multidisciplinary management, pediatric neurology

Pediatric neurological disorders account for a substantial proportion of the global burden of disease, contributing substantially to disability-adjusted life years through both premature mortality and years lived with disability (1). Unlike adult-onset conditions, neurological diseases in children arise within a dynamically developing nervous system, in which age-dependent neurobiological processes render neural circuits particularly vulnerable to injury and prone to dysmaturation, with enduring consequences for structural connectivity and functional outcomes (2, 3).

Among the most critical challenges in pediatric neurology are disorders emerging in the neonatal period—particularly those related to prematurity, neonatal seizures, and developmental and epileptic encephalopathies (DEE). Moreover, internal medical, metabolic, or acquired conditions (such as infections) may significantly influence neurological outcomes (4, 5).

Neonatal seizures represent the most common neurological emergency in the newborn period (4). Contemporary multicenter data indicate that a substantial proportion of neonatal seizures are electrographic-only and frequently associated with high seizure burden and early neurological morbidity (6). This electroclinical dissociation, together with marked etiological heterogeneity, complicates timely diagnosis and management (4).

Advances in neonatal intensive care have markedly improved survival among very preterm infants, shifting the clinical focus toward the mechanisms of white matter injury and subsequent brain dysmaturation that underlie long-term neurodevelopmental impairment (3). Within this framework, the encephalopathy of prematurity provides a unifying pathophysiological construct linking early-life seizures, disrupted network maturation, and adverse developmental trajectories (3, 7).

The molecular era has reshaped pediatric epileptology (8), leading to the revised ILAE classification, which formalized the concept of DEE and highlighted the intertwined contributions of genetic defects and epileptic activity to neurodevelopmental outcomes (9). Next-generation sequencing technologies have transformed the diagnostic landscape, and the rapid expansion of gene discovery in epileptic encephalopathies has significantly improved diagnostic yield in children with drug-resistant epilepsy and neurodevelopmental disorders (8, 12). Beyond epilepsy, the increasing identification of immune-mediated encephalopathies and genetically determined movement disorders further underscores the need for mechanism-based classification systems integrating molecular, immunological, and neurophysiological data (10, 11). Growing mechanistic insight has further revealed the substantial genetic and phenotypic heterogeneity of pediatric neurological disorders, with convergent electroclinical phenotypes arising from distinct molecular etiologies and, conversely, single-gene variants leading to markedly divergent clinical presentations (8, 9).

The extent of this variability becomes particularly apparent in immune-mediated encephalopathies. Fu et al. reported a pediatric case of anti-GABABR encephalitis presenting with seizures and neurological involvement, contributing to the characterization of this rare autoimmune condition in childhood. Expanding the spectrum of autoimmune encephalitis, Jiang et al. described myelin oligodendrocyte glycoprotein (MOG) antibody–associated encephalitis presenting despite initially normal magnetic resonance imaging, emphasizing that early neuroimaging findings may not reflect the underlying inflammatory process. Along the same spectrum, Liu et al. reported a pediatric case series of MOG antibody-associated encephalitis presenting as fluid-attenuated inversion recovery (FLAIR)-hyperintense cortical lesions in anti-MOG-associated encephalitis with seizures (FLAMES), defining a distinctive clinico-radiological phenotype characterized by unilateral cortical involvement. The authors highlighted that recognition of FLAMES can prevent misdiagnosis and unnecessary immunotherapy in children presenting with seizures without clear triggers. Further broadening the immunological landscape, Yamanaka et al. described the first clinical case of autoimmune encephalitis potentially associated with anti-neural cell adhesion molecule 1 (NCAM1) antibodies in a pediatric patient. This presentation closely mimicked anti-N-methyl-D-aspartate receptor encephalitis despite negative conventional antibodies panels, suggesting that additional immune targets may contribute to pediatric neuroinflammation. Together, these reports illustrate how autoimmune disorders in childhood may present with evolving and overlapping features, requiring careful longitudinal interpretation.

Diagnostic complexity in pediatric neurology is further amplified by children's limited ability to describe subjective symptoms. Clinical evaluation often relies on behavioral observation and age-dependent manifestations, increasing the risk of both under-recognition and misclassification. Minerva et al. reported a video-documented case of pediatric febrile myoclonus, a benign and poorly characterized condition that may be mistaken for epileptic or inflammatory disorders. By expanding its phenotypic spectrum and emphasizing careful clinical documentation, this contribution supports more accurate recognition of fever-associated paroxysmal movements and may help prevent unnecessary diagnostic procedures or antiseizure medications. Similarly, Rosen et al. examined the relationship between restless legs syndrome and growing pains, two common pediatric conditions with overlapping clinical features. By integrating physiological, genetic, and neurobiological evidence, the authors proposed that growing pains may represent an early manifestation within the restless legs spectrum, underscoring the need for refined diagnostic criteria and careful assessment of sleep-related and limb discomfort symptoms in children. They also highlighted the role of iron metabolism and dopaminergic pathways in explaining symptom overlap, providing a potential target for future intervention. These contributions highlight how similar clinical presentations may reflect distinct mechanisms, reinforcing the importance of nuanced differential diagnosis in early life.

Within this context of developmental variability and diagnostic uncertainty, precision-oriented strategies become essential. Advances in molecular genetics have significantly contributed to the etiological clarification of rare epileptic and neurodevelopmental conditions. Zhang et al. examined genotype–phenotype correlations in children with glucose transporter type 1 deficiency syndrome, documenting a broad spectrum of seizure types, ranging from generalized to focal manifestations, as well as diverse associated neurological features, including paroxysmal and persistent movement disorders. Wu et al. reported pathogenic and likely pathogenic NARS2 variants identified in pediatric patients with refractory epilepsy, expanding the mutational spectrum associated with this gene and further delineating the phenotypic heterogeneity linked with mitochondrial dysfunction. These studies also emphasized the importance of early genetic testing in guiding targeted metabolic therapies and counseling families about prognosis. Together, these studies underscore the increasing contribution of molecular genetics to the characterization of pediatric neurological disorders and epileptic encephalopathies, demonstrating how genetic investigation refines diagnostic definition while revealing biological mechanisms underlying clinical variability.

Beyond etiological identification, the search for biomarkers that capture disease activity, vulnerability, and long-term outcome is gaining relevance. Allen et al. examined systemic inflammatory responses in children with severe neurological impairment, revealing persistent alterations in cytokine reactivity, particularly in response to immune stimulation. By relating these inflammatory patterns to clinically relevant features such as infection burden and treatment complexity, this study provides mechanistic insight into ongoing neurological vulnerability and suggests that dysregulated inflammation may contribute to tertiary neurological injury and impaired responses to infection. At a broader level, Zhou et al. conducted a bibliometric and visualization analysis of therapeutic hypothermia in neonates. Rather than focusing on clinical efficacy, this study mapped the structure and evolution of the research landscape, identifying key contributors, thematic clusters, and emerging areas of interest, including neonatal seizures and long-term neurological outcomes. By delineating current research priorities and existing gaps, this contribution offers a contextual framework to guide future investigations aimed at refining neuroprotective strategies in neonatal populations.

Therapeutic approaches in pediatric neurology reflect the same complexity observed at the diagnostic level. Addressing established metabolic therapies, Hu et al. reviewed the clinical application of the ketogenic diet in children with DEE, summarizing evidence across different subtypes and highlighting key factors influencing therapeutic response. Extending metabolic strategies beyond dietary interventions, Cantillon et al. reported a phase 1/2a study assessing the safety and tolerability of CER-0001 (tricaprilin), an orally administered investigational ketogenic compound, in infants with drug-resistant epileptic spasms not receiving a ketogenic diet. By exploring a pharmacological approach designed to deliver ketogenic effects without dietary constraints, this early-phase investigation provides preliminary evidence supporting the feasibility of prescription metabolic therapy in a highly challenging clinical population. Both studies illustrate how metabolic interventions can be tailored to patient-specific phenotypes, offering individualized treatment options.

Among pharmacological strategies, increasing attention has been directed toward cannabidiol-based therapies across different clinical and experimental settings. Butera et al. reported an observational study evaluating cannabidiol as adjunctive therapy in a pediatric cohort with heterogeneous drug-resistant epilepsy. By considering not only seizure burden but also global clinical functioning, this contribution provides practical insights into the safety and tolerability of cannabidiol across a broad range of syndromes. Complementing these clinical observations, Pinto et al. examined the interaction between cannabidiol and phenobarbital in a neonatal rat seizure model using a pentylenetetrazole-induced paradigm. The study demonstrated dose-dependent potentiation of phenobarbital antiseizure effects by cannabidiol, offering experimental support for combination strategies while underscoring the need for careful assessment of long-term neurodevelopmental safety when introducing therapies during critical periods of brain maturation.

In the most severe epileptic encephalopathies, therapeutic management often requires integrated and time-sensitive interventions. Ding J.-M. et al. reported a pediatric case of new-onset refractory status epilepticus treated with prolonged high-dose intravenous esketamine, complicated by hemorrhagic cystitis. This case highlights a rare but potentially serious adverse effect and emphasizes the importance of monitoring cumulative toxicity in critically ill children. Caporale et al. described successful management of febrile infection–related epilepsy syndrome through a timely multimodal strategy integrating immunomodulatory treatment, ketogenic therapy, and induced hypothermia. These reports illustrate both the risks associated with aggressive rescue therapies and the potential benefits of coordinated, multimodal approaches in life-threatening pediatric conditions.

The clinical and therapeutic complexity outlined across these contributions underscores the need for structured models of care. Bakhos et al. explored the role of multidisciplinary clinics in the management of children with rare and medically complex epilepsies. Drawing on caregiver and clinician perspectives, this study highlights the benefits of coordinated, team-based care while identifying practical barriers to broader implementation. Similarly, Brisca et al. examined the impact of a newly established pediatric Intermediate Care Unit on the management of children with neuromuscular disorders. By bridging general pediatric wards and intensive care units, the Intermediate Care Unit reduced intensive care admissions and length of stay while maintaining appropriate monitoring, illustrating how organizational innovation can optimize resource allocation in neurologically fragile patients. Both studies underline that robust care structures are essential not only for acute management but also for integrating research and long-term follow-up.

Finally, long-term functional sequelae remain a significant source of morbidity in pediatric neurology. Ding Y. et al. evaluated the effectiveness and safety of sacral neuromodulation under general anesthesia in pediatric patients with neurogenic bladder. Beyond demonstrating meaningful clinical and urodynamic improvement, their study characterized heterogeneous recovery trajectories during mid-term follow-up, emphasizing the importance of individualized post-operative management and structured longitudinal assessment in chronic neurological conditions.

**Figure 1:**
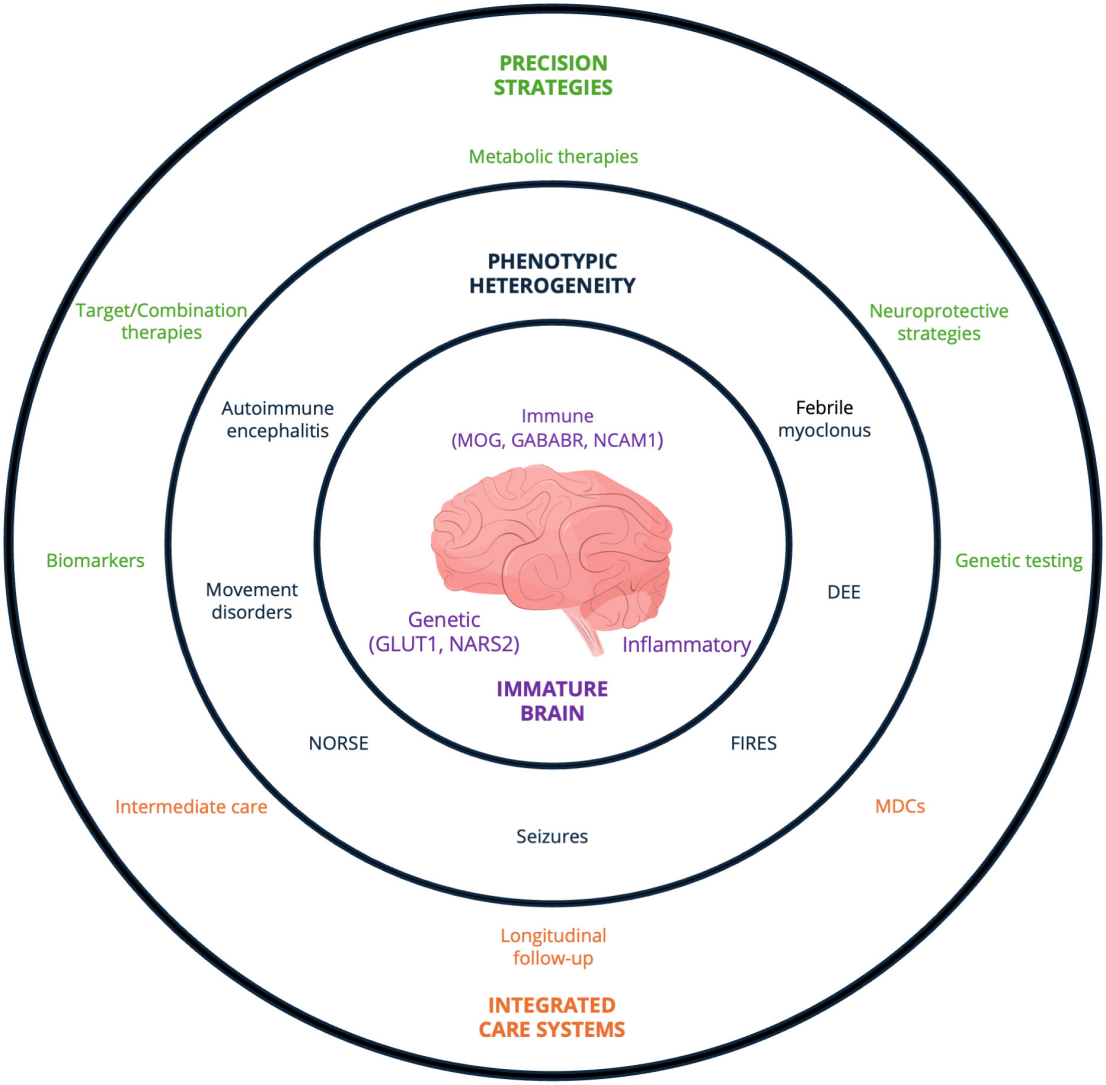
This concentric model integrates the principal themes emerging from the 19 contributions to this Research Topic, highlighting the necessity of bridging biological mechanisms, clinical heterogeneity, and organizational structures in pediatric neurology. It is structured around the immature brain as the biologically vulnerable substrate in which converging pathogenic mechanisms—genetic, immune, metabolic, and inflammatory—interact. The intermediate ring reflects the resulting phenotypic heterogeneity, whereby shared clinical presentations may arise from distinct biological substrates and, conversely, similar molecular mechanisms may manifest across divergent electroclinical phenotypes, including seizures, developmental and epileptic encephalopathies, autoimmune encephalitis, movement disorders, and severe refractory syndromes. The outer ring integrates precision-oriented diagnostic and therapeutic strategies alongside coordinated models of care, emphasizing the interplay between mechanism-based interventions and structured longitudinal management. DEE, developmental and epileptic encephalopathies; FIRES, Febrile infection-related epilepsy syndrome; MDCs, Multidisciplinary clinics; MOG, myelin oligodendrocyte glycoprotein; NCAM1, anti-neural cell adhesion molecule 1; NORSE, new-onset refractory status epilepticus.

Pediatric neurology is increasingly shaped by converging genetic, immune, metabolic, and developmental processes operating within the uniquely vulnerable context of the immature brain. In this evolving landscape, improving care for children with complex neurological disorders requires more than biological insight alone; it calls for precise diagnostics and biomarkers, innovative therapies, and coordinated multidisciplinary teams capable of translating scientific advances into tangible benefits for patients (see Figure 1). By integrating developmental, molecular, clinical, and organizational perspectives, the field can move toward truly precision-based pediatric neurology—one in which progress is defined not only by scientific discovery, but by its sustained impact across the continuum of early life.
